# Immunomodulatory Function of HBeAg Related to Short-Sighted Evolution, Transmissibility, and Clinical Manifestation of Hepatitis B Virus

**DOI:** 10.3389/fmicb.2018.02521

**Published:** 2018-10-24

**Authors:** Anna Kramvis, Evangelia-Georgia Kostaki, Angelos Hatzakis, Dimitrios Paraskevis

**Affiliations:** ^1^Hepatitis Virus Diversity Research Unit, Department of Internal Medicine, Faculty of Health Science, University of the Witwatersrand, Johannesburg, South Africa; ^2^Department of Hygiene, Epidemiology and Medical Statistics, Medical School, National and Kapodistrian University of Athens, Athens, Greece

**Keywords:** genotypes, subgenotypes, tolerogen, transmission, HBeAg seroconversion

## Abstract

Hepatitis B virus (HBV) infection, a global public health problem can be asymptomatic, acute or chronic and can lead to serious consequences of infection, including cirrhosis, and hepatocellular carcinoma. HBV, a partially double stranded DNA virus, belongs to the family *Hepadnaviridae*, and replicates via reverse transcription of an RNA intermediate. This reverse transcription is catalyzed by a virus-encoded polymerase that lacks proof reading ability, which leads to sequence heterogeneity. HBV is classified into nine genotypes and at least 35 subgenotypes, which may be characterized by distinct geographical distributions. This HBV diversification and distinct geographical distribution has been proposed to be the result of the co-expansion of HBV with modern humans, after their out-of-Africa migration. HBeAg is a non-particulate protein of HBV that has immunomodulatory properties as a tolerogen that allows the virus to establish HBV infection *in vivo*. During the natural course of infection, there is seroconversion from a HBeAg-positive phase to a HBeAg-negative, anti-HBe-positive phase. During this seroconversion, there is loss of tolerance to infection and immune escape-HBeAg-negative mutants can be selected in response to the host immune response. The different genotypes and, in some cases, subgenotypes develop different mutations that can affect HBeAg expression at the transcriptional, translational and post-translational levels. The ability to develop mutations, affecting HBeAg expression, can influence the length of the HBeAg-positive phase, which is important in determining both the mode of transmission and the clinical course of HBV infection. Thus, the different genotypes/subgenotypes have evolved in such a way that they exhibit different modes of transmission and clinical manifestation of infection. Loss of HBeAg may be a sign of short-sighted evolution because there is loss of tolerogenic ability of HBeAg and HBeAg-negative virions are less transmissible. Depending on their ability to lead to HBeAg seroconversion, the genotype/subgenotypes exhibit varying degrees of short-sighted evolution. The “arms race” between HBV and the immune response to HBeAg is multifaceted and its elucidation intricate, with transmissibility and persistence being important for the survival of the virus. We attempt to shed some light on this complex interplay between host and virus.

Hepatitis B virus (HBV), which belongs to the family *Hepadnaviridae* is the smallest DNA virus infecting man. HBV has a partially double-stranded, circular DNA genome of ~3,200 base pairs, with four overlapping reading frames (ORFs). HBV replicates via an RNA intermediate, through the process of reverse transcription catalyzed by the viral polymerase that lacks proof reading ability. Thus, sequence heterogeneity is a feature of the virus and within the host HBV exists as a quasispecies of mixed viral strains. Based on an intergroup divergence of >7.5% across the complete genome, HBV has been classified phylogenetically into 9 genotypes, A–I (Norder et al., 2004; Kramvis et al., 2005; Yu et al., 2010; Kramvis, 2014) (Figure 1), with a putative 10th genotype, “J,” isolated from a single individual (Tatematsu et al., 2009). Genotypes A–D, F, H, and I are classified further into at least 35 subgenotypes, using between ~4 and 8% intergroup nucleotide divergence across the complete genome and good bootstrap support, (Norder et al., 2004; Kramvis et al., 2005, 2008; Kramvis, 2014). The genotypes, and in some cases the subgenotypes, have distinct global and local geographical distributions, with the genotypes, prevailing in the two regions where HBV is endemic, south East Asia, and Africa, being different (Figure 2) (Kramvis et al., 2005; Kramvis, 2014). This HBV diversification and distinct geographical distribution has been proposed to be the result of the co-expansion of HBV with modern humans, after their out-of-Africa migration (Paraskevis et al., 2013).

**Figure 1:**
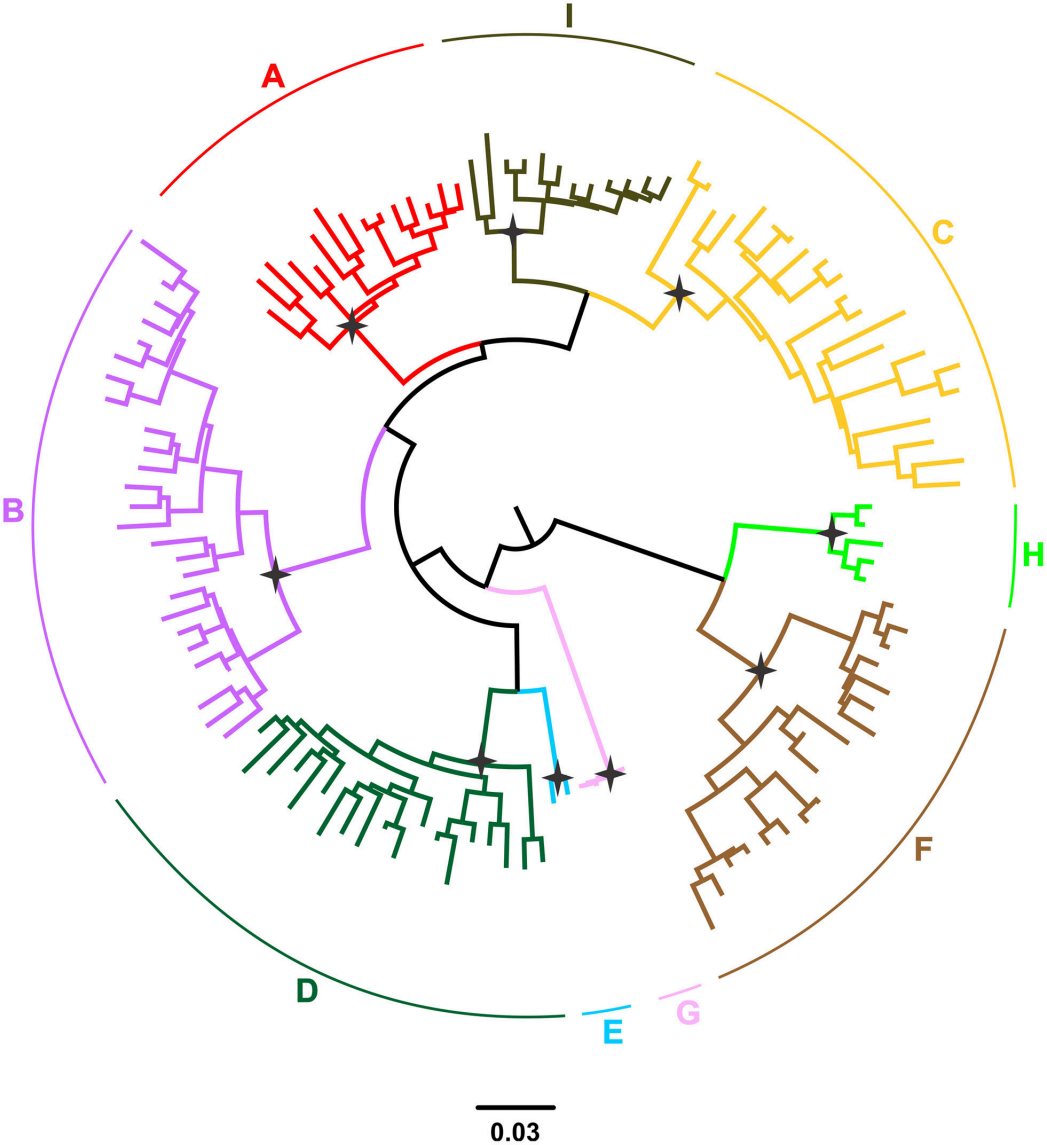
Phylogeny of the HBV major clades **(A–I)** estimated by FastTree v2.1. The phylogenetic tree was inferred using publically available, full-length genomic sequences. Genotypes are shown in different colors. The names of genotypes are shown on the top of the corresponding clades. Selected nodes with SH value equal to 1 are denoted by stars.

**Figure 2:**
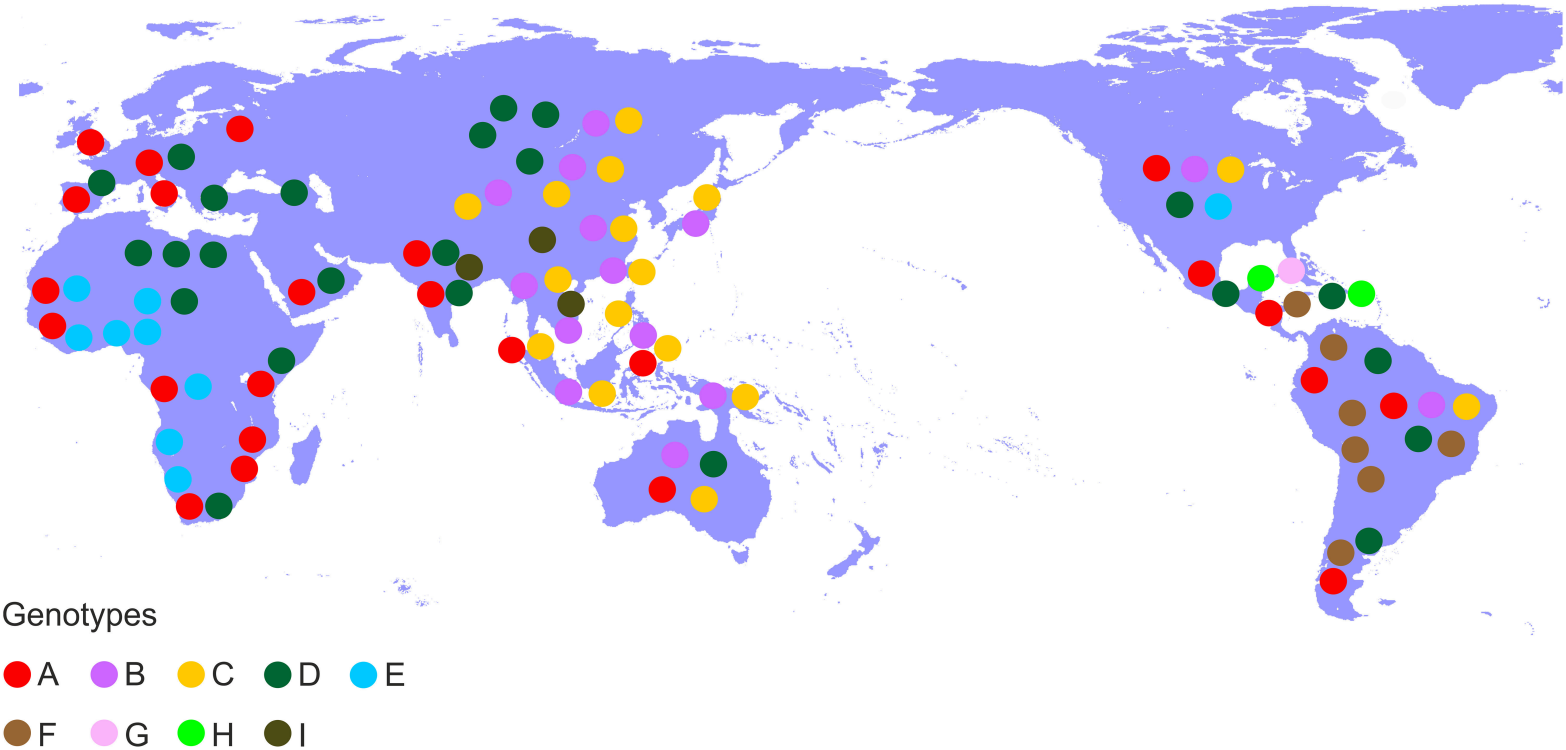
Global distribution of the HBV genotypes. Genotypes are shown in coloured disks in the geographical regions of their prevalence.

In addition to encoding for the structural proteins, HBcAg (core or capsid protein) and the viral envelope proteins [three forms of HBsAg, small (S), middle (M), and large (L)] and the polymerase/reverse transcriptase, the compact genome of HBV encodes for two non-particulate proteins, the X protein (a transcriptional transactivator) and HBeAg (Tiollais et al., 1981). Antibodies are directed against both structural and non-structural proteins of HBV. Anti-HBc is non-neutralizing and a sign of exposure to HBV. Anti-HBs is the neutralizing antibody, directed against “a” dominant epitope of the viral envelope and anti-HBs levels >10 IU/L, following either natural infection or vaccination, are indicators of immunity. Anti-HBe is directed against B cell epitopes shared by both HBeAg and HBcAg, with HBeAg acting as a decoy for HBcAg. HBcAg and HBeAg T cell epitopes are also cross-reactive in both humans (Ferrari et al., 1991) and mice (Milich et al., 1987). Anti-HBe seroconversion can occur up to 6 years before the actual loss of HBeAg or the onset of liver damage (Thompson et al., 2007).

The complex interplay between the host and viral factors (including genotype/subgenotype, viral load and HBeAg status) play an important role in determining the clinical outcomes of HBV infection. HBV infection can be asymptomatic, acute, chronic (HBsAg-positive for longer than 6 months), which can lead to serious consequences of infection, including cirrhosis, and hepatocellular carcinoma (HCC; liver cancer). HBV is generally non-cytopathic. The liver damage associated with either acute or chronic hepatitis B is as a result of the immune response attack on hepatocytes, in a bid to eliminate HBV during the immune clearance or reactive phase, which leads to necroinflammation. In order to overcome the effects of the immune response viruses can code for immunomodulatory proteins, such as HBeAg in the case of HBV, and also evolve genetically in order to escape the immune response. Immune escape mutants can be selected during the course of natural infection in response to the host immune response. In HBV the complex patterns of purifying selection are as a result of the overlapping ORFs (Mizokami et al., 1997) and the high frequency of recombination (Simmonds and Midgley, 2005; Zhou and Holmes, 2007). The “arms race” between HBV and the immune response is multifaceted and its elucidation intricate, with transmissibility and persistence being important for the survival of the virus. Here an attempt will be made to shed some light on this complex interplay.

## HBeAg as an immunomodulator

The 25 kDa precursor of HBeAg, with an additional 29 amino acids on its amino terminus relative to HBcAg, is translated from the *preC/C* ORF on the precore mRNA [1901–2452/2488 from the *Eco*RI site, (Kramvis et al., 2005)] (Summers and Mason, 1982; Messageot et al., 2003). HBcAg is translated from the pregenomic RNA from position 1901. The basic core promoter (BCP) of HBV controls the transcription of the *preC/C* region from both transcripts (Yuh et al., 1992; Yu and Mertz, 1996) (reviewed in Kramvis and Kew, 1999). The amino terminal signal peptide directs the precursor to the endoplasmic reticulum (ER), where it is post-translationally modified by cleavage on the amino and carboxyl termini to give rise to the mature HBeAg that is expressed in the hepatocyte cytosol and also secreted in its soluble form in the serum (Revill et al., 2010).

Even though its exact function has not been determined, the conservation of HBeAg in all hepadnaviruses signifies an important role of this non-particulate secreted protein (Revill et al., 2010). Moreover, HBeAg must impart an evolutionary advantage to HBV because even though HBeAg-negative mutant strains of HBV exist (see below), they have not replaced the wild-type virus. From various animal studies it is evident that HBeAg is not involved in viral infection, replication, and assembly (Chang et al., 1987; Schlicht et al., 1987; Chen et al., 1992; Milich and Liang, 2003), but is important for natural infection *in vivo* (Milich and Liang, 2003). It is thought that the virus has retained the secretory form of HBcAg because it has immunomodulatory functions. HBeAg downregulates the immune response to HBcAg by deletional, nondeletional, central, and peripheral immune tolerance (Milich et al., 1990, 1998; Milich, 1997; Milich and Liang, 2003; Chen et al., 2004, 2005). Thus, HBeAg-mediated immune regulation may predispose to chronicity and persistence following *in utero* or perinatal transmission and prevent severe liver injury during adult infections (Milich and Liang, 2003). Clinically, HBeAg is an index of viral replication, infectivity, inflammation, severity of disease and response to antiviral therapy.

HBeAg is expressed by both human and non-human hepadnaviruses, with all mammalian-infecting sequences showing high conservation within the amino terminus of the precursor and key immunomodulatory epitopes (Revill et al., 2010), including B-cell epitopes and T-cell recognition sites in the region that overlaps with HBcAg (Milich et al., 1987; Belnap et al., 2003; Billaud et al., 2005). Thus, HBeAg:

- *elicits both humoral and cell-mediated immunity,which differ from that directed to HBcAg* (Huang et al., 2006)

HBcAg and HBeAg share extensive amino acid homology but differ in their structure and localization. HBcAg is particulate and found in the cell whereas HBeAg is non-particulate/monomeric and can be found in the cell but is also secreted extracellularly (Milich and Liang, 2003). Because of these differences they are targeted by different antibodies (Imai et al., 1982). The antibody to HBcAg can either be T-cell dependent or independent and the non-cross-reactive antibody to HBcAg is greater than that to that directed against HBeAg (Milich and McLachlan, 1986). HBeAg exhibits low T-cell dependent antibody response (Milich and Liang, 2003). The CD8^+^ T-cell (CTL) response against HBcAg is important in HBV elimination in humans (Bertoletti et al., 1991; Chisari and Ferrari, 1995). HBcAg and HBeAg are highly cross-reactive at the CD4^+^ and CD8^+^ T-cell levels (Milich et al., 1988; Bertoletti et al., 1991; Kuhrober et al., 1997; Townsend et al., 1997) and are indistinguishable in terms of CTL priming and CTL target recognition *in vitro* (Kuhrober et al., 1997; Townsend et al., 1997; Frelin et al., 2009). HBeAg expressed in hepatocytes *in vivo* is a superior target for HBcAg/HBeAg CTLs compared to HBcAg alone (Frelin et al., 2009). In order to be recognized by the CTLs, cytosolic HBeAg and/or its precursors must be processed and presented in the context of major histocompatibility complex (MHC) class I molecules (Frelin et al., 2009), whereas HBeAg presentation for CD4^+^ cells is via the MHC class II pathway (Milich and Liang, 2003).

- *functions as a T cell tolerogen* (Chen et al., 2005) *and regulates the immune response against the intracellular nucleocapsid* (Chen et al., 2004)

An important function of the non-particulate HBeAg, which is the only HBV protein to cross the placenta (Milich and Liang, 2003), is to establish neonatal T cell tolerance to both HBeAg and HBcAg (Milich et al., 1990; Hsu et al., 1992; Chen et al., 2004). Therefore, it is essential that HBeAg remains non-particulate so that it can cross the placenta (Schodel et al., 1993).

- *regulates the innate immune response to viral infection within hepatocytes*.

The adaptive immune response is attenuated, by the preferential activation of Th2 cells over Th1 cells, following NF-kB activation by HBeAg (Yang et al., 2006). Th2 cells induce a non-protective humoral response whereas Th1 cells stimulate macrophages, which eliminate virions (Forsthuber et al., 1996; Huang et al., 2006). HBeAg in the serum leads to a switch from the normal Th1-mediated anti-HBc antibody response to Th2 phenotype (Milich et al., 1998). The difference in the activation of the different Th cell subsets is probably because the primary antigen-presenting cells are different for HBcAg and HBeAg, being B cells for the former and dendritic/macrophage cells for the latter (Milich et al., 1997) This preferential activation by HBeAg of Th2 cells, together with the depletion of Th1 cells, which are required for viral clearance, can lead to the persistence of HBV. HBeAg also down-regulates the expression of the Toll-like receptor (TLR) family within hepatocytes (Riordan et al., 2003; Visvanathan et al., 2007). When peripheral blood monocyte cells, were pre-treated with HBeAg, they displayed an impaired TLR signaling response (Visvanathan et al., 2007).

## HBeAg to anti-HBe seroconversion

Broadly, the natural history of HBV can be divided into at least four phases, which are differentiated by the level of viral replication and the host immune response:

- The **high replicative, low inflammatory phase** (previously immune tolerant phase) (Gish et al., 2015) is HBsAg/HBeAg-positive, with high HBV DNA levels (>2 × 10^5^ IU/ml) (Ni et al., 2007), with minimal liver inflammation, normal liver enzyme levels and asymptomatic(Chang et al., 1988). HBV does not select mutations frequently.
- The **immune clearance or reactive phase** is characterized by fluctuating alanine amino transferase (ALT) and HBV DNA levels and ending with spontaneous HBeAg loss (Ni et al., 2007; Liaw and Chu, 2009). HBeAg seroconversion is accompanied by elevated ALT and decreased HBV DNA levels (Ni et al., 2007). Core and precore mutations emerge in HBV.
- The **HBeAg-negative chronic hepatitis phase**(Gish et al., 2015), characterized by necroinflammation with high or fluctuating ALT levels and unsuccessful immune clearance, moderate to high viral loads and progressive liver disease.
- The **low replicative phase** (previously “inactive HBsAg-positive carrier” phase)(Gish et al., 2015) (post-HBeAg seroconversion). Although like the HBeAg-negative chronic hepatitis phase, this phase is also characterized by the absence of HBeAg, anti-HBe positivity, ALT levels are normal and HBV DNA levels are low or undetectable (<2 × 10^3^ IU/ml).

The length of the HBeAg-positive phase is important in determining both the mode of transmission and the clinical course of HBV infection. Mother-to-child transmission and subsequent chronic infection in the infant are favoured when mothers are HBeAg-positive (Chen et al., 2012), whereas children born to HBeAg-negative mothers are more likely to develop acute hepatitis B or may be infected horizontally later in life, if not immunized (Hadziyannis and Vassilopoulos, 2001; Kramvis, 2016). Following acute HBV infection, the development of chronic infection requires the expression of HBeAg (Hadziyannis, 2011) with a longer duration of HBeAg-positivity, increasing the risk of liver disease progression (Chu and Liaw, 2007). Although HBeAg loss is universally considered to be a favourable outcome (Hsu et al., 2002; Chu and Liaw, 2007), usually accompanied by decreased viral load and clinical remission, active hepatitis, cirrhosis, and hepatocellular carcinoma can develop in a minority of patients even after seroconversion (Hsu et al., 2002).

HBeAg seroconversion is a dynamic process lasting a number of years and involves the interaction between the host immune response and changes in the quasispecies and viral loads of HBV (Lim et al., 2007).

## Viral evolution and emergence of mutants affecting HBeAg expression

The loss of HBeAg between the high replicative, low inflammatory phase and HBeAg-negative chronic hepatitis phase is accompanied by:

- Decrease in viral sequence diversity

Viral sequence diversity was found to be 2.4-fold higher in both spontaneous and interferon-induced HBeAg seroconverters compared with non-seroconverters (Lim et al., 2007). Mutations, in both the HBeAg and HBcAg, were positively selected in 70% of the seroconverters compared to 5% of the non-seroconverters (Lim et al., 2007).

- Increase in viral substitution rates

Viral substitution rates have been shown to increase between the high replicative, low inflammatory phase and the immune clearance/reactive phase (Hannoun et al., 2000). Following seroconversion, immune pressure continues to drive viral mutagenesis even though the viral loads may be low (Lim et al., 2007) and HBV replicates less efficiently in the HBeAg-negative phase of disease (Volz et al., 2007).

- Emergence of HBeAg-negative mutants

The viral quasispecies following seroconversion are defective in HBeAg production as a result of mutations in the BCP and *preC/C* ORF. Mutations in the BCP and *preC* can influence the expression of HBeAg at the transcriptional, translational and post-translational levels (Kramvis, 2016) (Table 1).

**Table 1:** Mutations affecting HBeAg expression.

**Level of effect**	**Genomic region**	**Mutation**	Regression coefficient^*^ **(Kramvis et al.**, **2008****)**		**References**
			**Positive**	**Negative**	
Genotype/subgenotype variant	*preC*	1858C	A (3.04) C2 (2.59) F (1.49) F2 (1.95) H (1.48)	B (−1.73) C (−0.55) C1 (−2.34) D (−2.39) E (−1.68) F1 (−1.95)	Kramvis et al., 2008
Transcriptional	BCP	1762T1764A	C (0,89) G (1.80)	B (−0.92) F (−0.78)	Buckwold et al., 1996
Translational	BCP	Kozak 1809–1812	A1		Ahn et al., 2003
	*preC*	Precore start codon 1817TAA	G (3.64)	D(−2.12)	
	*preC*	G1896A	B (0.89) D (0.97) G	C (−0.86) E (−1.42)	Carman et al., 1989
Post-translational	*preC*	G1862T	A1		Chen et al., 2008; Bhoola and Kramvis, 2017

* *The logistic regression method was used to identify HBV mutations associated with specific genotypes/ subgenotypes (Afifi et al., 2004). Larger values show tighter correlations, with positive values related to a positive association, and negative values to a negative association. All associations were statistically significant using 2-sided p-values (Kramvis et al., 2008)*.

**Figure 3:**
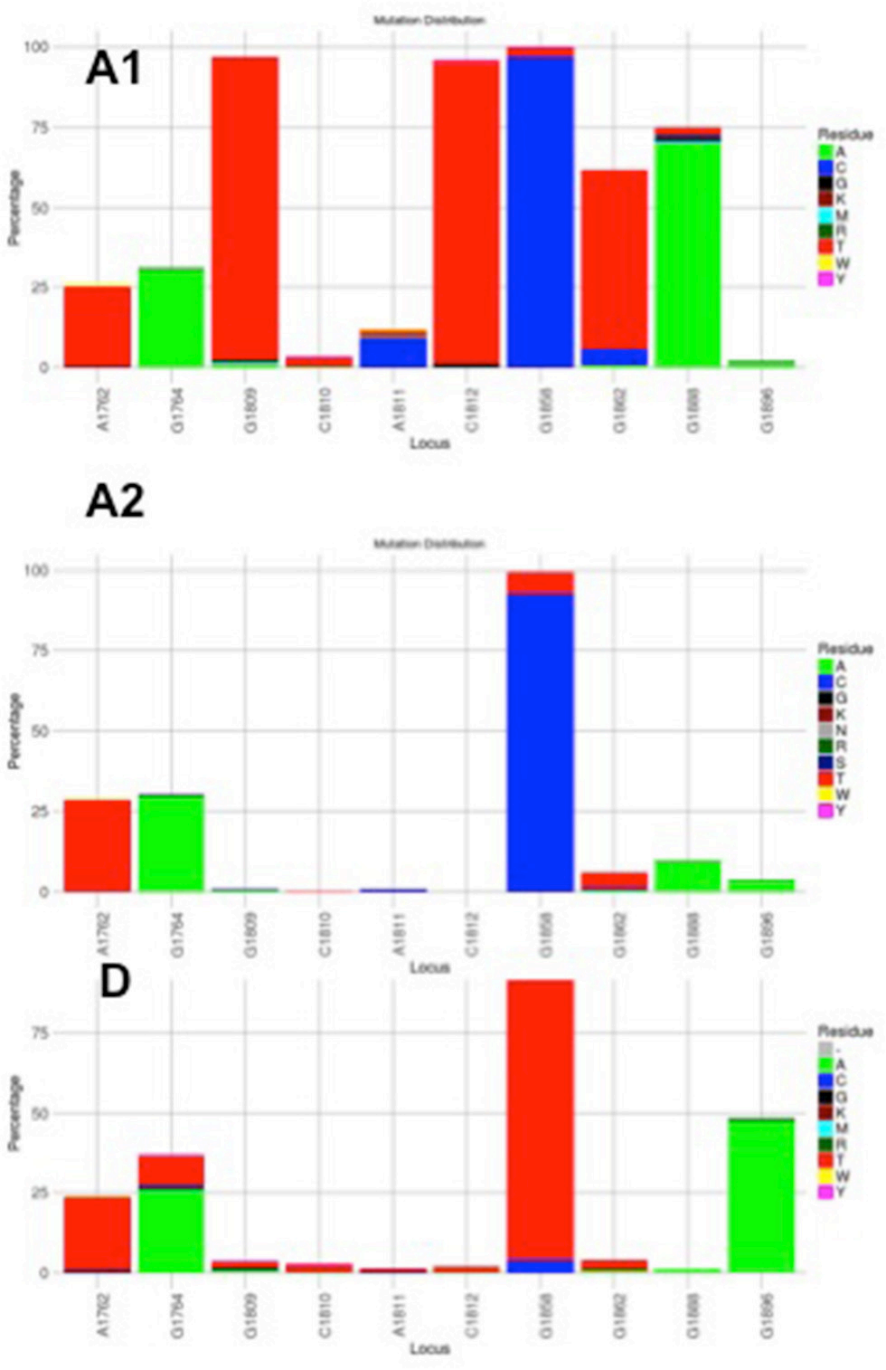
Mutation distribution graphs generated using the Mutation Reporter Tool (Bell and Kramvis, 2013) showing the percentage of mutant residues relative to the reference motif found at the ten loci of interest specified (1762, 1764, 1809-1812, 1858, 1862, 1888, 1896). Three data sets were submitted to the tool to produce the three graphs showing the mutation distribution for 605 subgenotype A1, 730 subgenotype A2 and, 1899 genotype D unselected sequences. The basic core promoter/precore sequences (1750 – 1900 from *Eco*R1 site) was downloaded from http://hvdr.bioinf.wits.ac.za/alignments (Bell et al., 2016). The reference motif used was AGGCACGGGG. This is also shown by the letter preceding each locus on the X-axis. To facilitate direct comparisons between the graphs, conserved loci were not suppressed and the Y-axis was scaled to 100% by selecting the appropriate controls on the input page.

Position 1858 in the preC/C can be either a C or T (Figure 3) and is differentially associated with the different genotypes/subgenotypes (Table 1). This variation at position 1858 can influence, which of the mutations, affecting HBeAg expression, will develop (Kramvis et al., 2008) and their frequency (Revill et al., 2010).

Although the HBV sequences deposited in the public databases, such as GenBank, may not be entirely representative of the strains circulating globally, they provide the best available datasets of genotyped strains of HBV (Bell et al., 2016). From this data set it is evident that the genotype can affect the frequency of the BCP/PC mutations. The BCP double mutation 1762T/1764A occurs in ~25% of downloaded sequences (Bell et al., 2016) without difference between subgenotypes A1, A2, and genotype D (Figure 3). In two case control studies, independently of HBeAg status, 1762T/1764A was significantly more frequent in genotypes A and C compared to D and B, respectively (Orito et al., 2001; Tanaka et al., 2004). A mutation, which leads to a stop codon that truncates HBeAg during translation, G1896A, does not develop in the genotypes/subgenotypes in which 1858C occurs frequently (Li et al., 1993; Lok et al., 1994) and in which 1858C is positively associated with, namely, A, C2, F2, and H (Kramvis et al., 2008). On the other hand, G1896A is frequent in genotypes/subgenotypes, which have 1858T, C1, D, E, and F (Kramvis et al., 2008; Revill et al., 2010). As illustrated in Figure 3 more than 40% of genotype D sequences downloaded from Genbank had G1896A whereas it was found in very few genotype A sequences. Overall G1896A occurs in up to 30% of all sequences, most frequently detected in genotypes G (100%), and B (50%), followed by genotypes D (40%) and C (23%) (Revill et al., 2010). All genotype G and the majority of subgenotype B6 sequences encode this mutation and it is least frequently observed in genotype A (1.5%) and genotype F (5%) (Revill et al., 2010). Thus, because of these differences the estimated annual rate of HBeAg to anti-HBeAg seroconversion can differ between the genotypes. Genotype B has higher annual rate of seroconversion of 15.5% compared to genotype C (7.9%) (Kao et al., 2004) and E (7.4%) (Shimakawa et al., 2016). In a Taiwanese study, it was shown that HBeAg seroconversion of patients infected with genotype C occurred 10 years later than those infected with genotype B (Kao et al., 2004).

**Figure 4:**
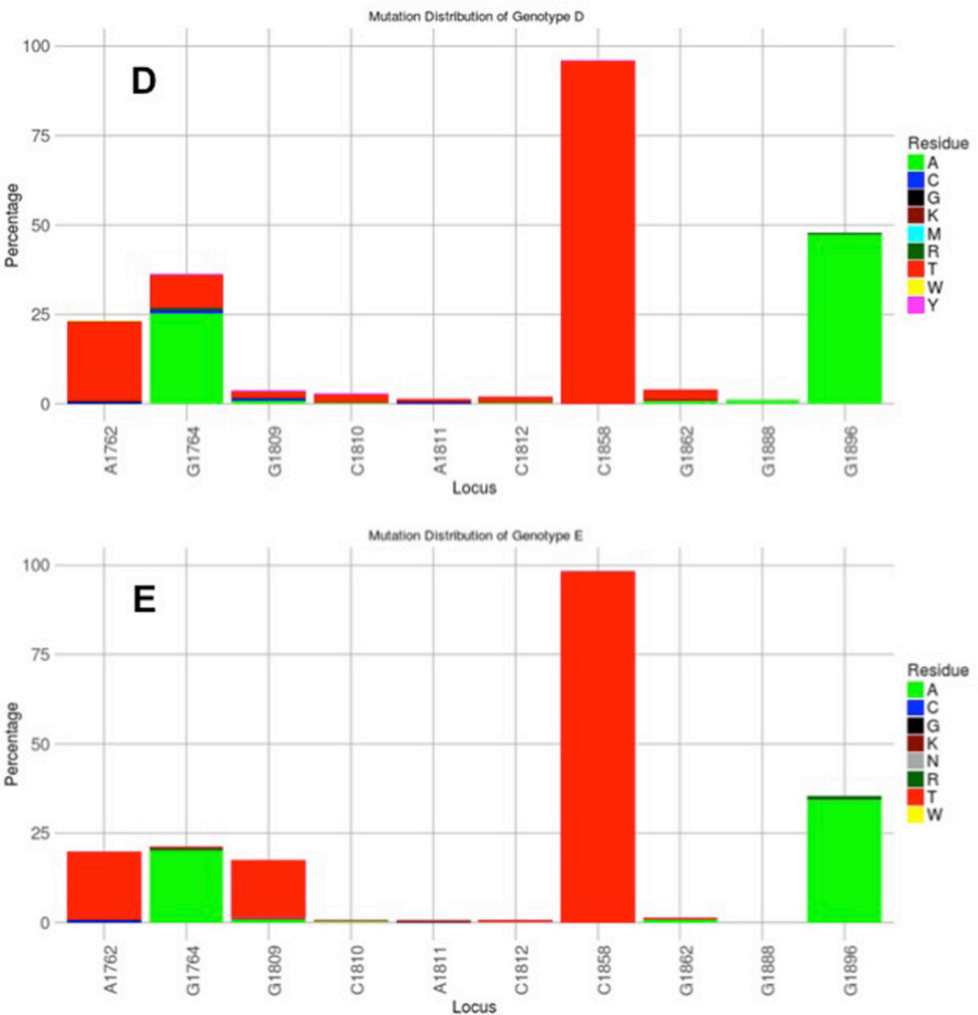
Mutation distribution graphs generated using the Mutation Reporter Tool (Bell and Kramvis, 2013) showing the percentage of mutant residues relative to the reference motif found at the ten loci of interest specified (1762, 1764, 1809-1812, 1858, 1862, 1888, 1896). Two data sets were submitted to the tool to produce the two graphs showing the mutation distribution for 1899 genotype D and 471 genotype E unselected sequences. The basic core promoter/precore sequences (1750–1900 from *Eco*R1 site) was downloaded from http://hvdr.bioinf.wits.ac.za/alignments (Bell et al., 2016). The reference motif used was AGGCACGGGG. This is also shown by the letter preceding each locus on the X-axis. To facilitate direct comparisons between the graphs, conserved loci were not suppressed and the Y-axis was scaled to 100% by selecting the appropriate controls on the input page.

Subgenotype A2 does not select the G1896A mutation, nor the other mutations found in subgenotype A1 that contribute to the HBeAg-negativity of this unique strain (Table 1). In particular, the mutations in the Kozak sequence upstream from the precore start codon (1809–1812), characteristic of subgenotype A1 (Baptista et al., 1999; Kramvis and Kew, 2007; Revill et al., 2010), interfere with translation of HBeAg by a leaky scanning mechanism (Ahn et al., 2003), do not occur in subgenotype A2. Therefore, generally subgenotype A2 has a high frequency of HBeAg-positivity compared to subgenotype A1 and this is statistically significant in individuals younger than 30 years (Tanaka et al., 2004). The reason for the high frequency of HBeAg-positivity in subgenotype A2 is possibly because the only mutation that is selected in A2 that can affect HBeAg expression is 1762T/1764A. This double mutation is found more frequently in HBeAg-negative carriers of subgenotype A2 HBV, whereas, the frequency of 1762T/1764A is not affected by HBeAg status in subgenotype A1 (Tanaka et al., 2004). In genotype D isolates from HBeAg-negative individuals, both 1762T1764A and 1896A were more frequent than from HBeAg-positive individuals (Tanaka et al., 2004). The BCP 1762T/1764A mutations are not selected in subgenotype B6 strains, prevalent in people living in Canadian Arctic and who are frequently HBeAg-negative, as a result of 1896A and precore start codon mutations (Osiowy et al., 2011). Even though genotypes D and E cannot be differentiated in the precore region and both have 1858T, they do not develop G1896A at the same frequency. In Sudan, where genotypes D and E co-circulate viral loads were significantly higher in genotype E-infected patients compared to genotype D-infected, with patients infected with genotype E, showing a significantly higher frequency of HBeAg-positivity in blood donors (Mahgoub et al., 2011), asymptomatic carriers and liver disease patients (Yousif et al., 2013). The high frequency of HBeAg-negativity in genotype D was as a result of G1896A (Mahgoub et al., 2011; Yousif et al., 2013), which correlates with the statistically positive and negative association of G1896A, with genotype D and E, respectively (Kramvis et al., 2008) (Table 1). This is confirmed by a comparison of the frequency of G1896A in sequences downloaded from the public databases (Bell et al., 2016), where G1896A occurred 47.2% of genotype D compared to 34.2% genotype E sequences (*p* < 0.0001) (Figure 4). Genotype G is unique in that all sequences have a premature stop codon at position 2 of the precore precursor protein and therefore HBeAg is not expressed (Stuyver et al., 2000).

Subgenotype A1 is the most “sophisticated” in terms of its control of HBeAg expression (Kramvis and Kew, 2007). Firstly, like all other genotypes and subgenotype A2, it can develop 1762T/1764A. Secondly, instead of Kozak 1809–1812 GCAC, which is present in subgenotypes A2 and D3, subgenotype A1 has TCAT (Kramvis and Kew, 2007). These variations are characteristic of subgenotype A1 and affect HBeAg expression at the translational level (Ahn et al., 2003), by converting the Kozak region from an optimal to a suboptimal translation context (Kimbi et al., 2004) and, causing decreased translation of HBeAg by a ribosomal leaky scanning mechanism (Ahn et al., 2003). Compared to subgenotypes A2 and D3, transfection with subgenotype A1 led to a lower expression of the precore/core precursor in the secretory pathway and a higher co-localization in the nucleus (Bhoola and Kramvis, 2016). This reduction in HBeAg levels is comparable to that observed in the presence of 1762T/1764A and when occurring together, the Kozak and BCP 1762T/1764A mutations reduce HBeAg expression in an additive manner (Ahn et al., 2003). Thirdly, a G to T transversion at position 1862 in the precore region of HBV occurs more frequently in subgenotype A1 isolates from HBeAg-negative than in HBeAg-positive South Africans (Kramvis et al., 1997, 1998) and affects HBeAg expression at the post-translational level (Sugauchi et al., 2004; Chen et al., 2008; Inoue et al., 2009; Bhoola and Kramvis, 2017) This mutation results in a valine to phenylalanine substitution at the−3 position of the signal peptide cleavage site at position 19 of the precursor protein. Phenylalanine interferes with signal peptide cleavage (Nielsen et al., 1997). This leads to decreased HBeAg expression as a result of the increased retention of the precursor in the cytoplasm of the hepatocyte (Chen et al., 2008; Inoue et al., 2009; Bhoola and Kramvis, 2017). When this mutation was introduced into a genotype D plasmid driven by a cytomegalovirus promoter, it resulted in a 54% reduction in the secretion of HBeAg relative to the wild-type and to the formation of aggresomes (Chen et al., 2008). When this mutation was introduced into a subgenotype A1 backbone, the decrease in expression of secreted HBeAg was less than that in the genotype D context (22%). The mutant was found to lead to the accumulation of the HBeAg precursor protein in the ER and ER-Golgi intermediate compartment (ERGIC). This accumulation resulted in an earlier activation of the three UPR pathways, but not to an increase in apoptosis (Bhoola and Kramvis, 2016).

Thus, in the various genotypes and subgenotypes, HBeAg loss, and immune response escape are as a result of a number of mutations. In order to survive, HBV has to balance its ability to establish an infection (HBeAg-positive, immunotolerogenic phase) and escape the immune response (HBeAg-negative phase) in order to persist.

## Interaction of the virus and host

Previous studies have shown that a few years before seroconversion and as soon as the levels of HBeAg begin declining, the genetic diversity of the virus increases (Hannoun et al., 2000; Lim et al., 2007; Cheng et al., 2013). Notably viral diversity does not follow this trend in non-seroconverters but remains at the low-level seen over the course of the immunotolerant phase (Lim et al., 2007; Wu et al., 2011; Cheng et al., 2013). Given the stable rate at which mutations accumulate in the HBV genome during the different stages of HBV replication, the differences in viral diversity observed, firstly between seroconverters and non-seroconverters, during the course of the infection and close to the time of seroconversion are because of the strong selective pressure of the immune response (Cheng et al., 2013). Specifically, as soon as the levels of HBeAg are decreasing, tolerance diminishes, leading to increased immune response and the selection of escape variants, which are infrequently selected during the immunotolerant phase. The increased genetic diversity is inversely related to HBV-DNA levels, intimating that the variants selected before and during the HBeAg negative phase have a lower replicative capacity than the virions circulating in the absence of immune selective pressure (Cheng et al., 2013). Thus, the intra-host viral evolution also follows different stages according to the HBeAg and immune-status of the host. During the immunotolerant phase, the virus replicates without constraint from the host and thus there is no need to select multiple variants; in contrast, during the immune clearance and seroconversion phase, different variants are selected because of the strong selective pressure (Lim et al., 2007; Cheng et al., 2013). Interestingly, during the latter phase, multiple variants with higher genetic variability in the non-overlapping genomic regions are selected and circulate.

Therefore, there is a complex interaction of the virus and the host. As it has been previously found, HBV has co-expanded with modern humans for at least 28,000 years suggesting a long period of interaction (Paraskevis et al., 2013). HBV major clades (genotypes) have been generated as a result of founder effects of different strains spreading in distinct geographic regions. Similarly, subgenotypes, belonging within HBV major clades, are the result of more recent events of strains. The time of the most common recent origin (tMRCA) of different genotypes has been previously estimated to occur several 1,000 years ago (Paraskevis et al., 2013), while the tMRCA of the subgenotypes is more recent (Paraskevis et al., 2013). Given that HBV has co-expanded with humans for a long time and also that some genotypes have been more frequently found in specific areas and populations (i.e., F/H in indigenous populations in America; B and C in Asia; many different subgenotypes have been infecting mostly indigenous populations across the globe) suggests that some of the genomic characteristics related to the HBeAg expression probably have been shaped over a long time period. This is presently a hypothesis and concrete evidence will come from the analysis of HBV from ancient samples.

Stochastic mutations lead to the break of immune tolerance and/or increased immune reactivity, which drives viral evolution from a low- to a high-level positive selection stage that in turn leads to decreased HBV DNA levels because of increased immune pressure and less efficient viral replication. This increased sequence diversity in the intra-host quasispecies in seroconverters prior to the loss of HBeAg was found to be largely as a result of sequence variation in the precore/core open reading frame and its regulatory elements (Harrison et al., 2011). This sequence variation in the region encoding HBeAg can influence the immunomodulatory role of HBeAg, the ability of HBV to persist and be transmitted, as well as its virulence. The driving forces of viral evolution during the different stages of HBV infection are complex and difficult to present in a linear fashion. The balance and trade-offs of the immunomodulation and immune escape of HBeAg can be influenced by the different genotypes/subgenotypes.

## Short sighted evolution, transmissibility and clinical manifestation related to HBeAg expression

**Figure 5:**
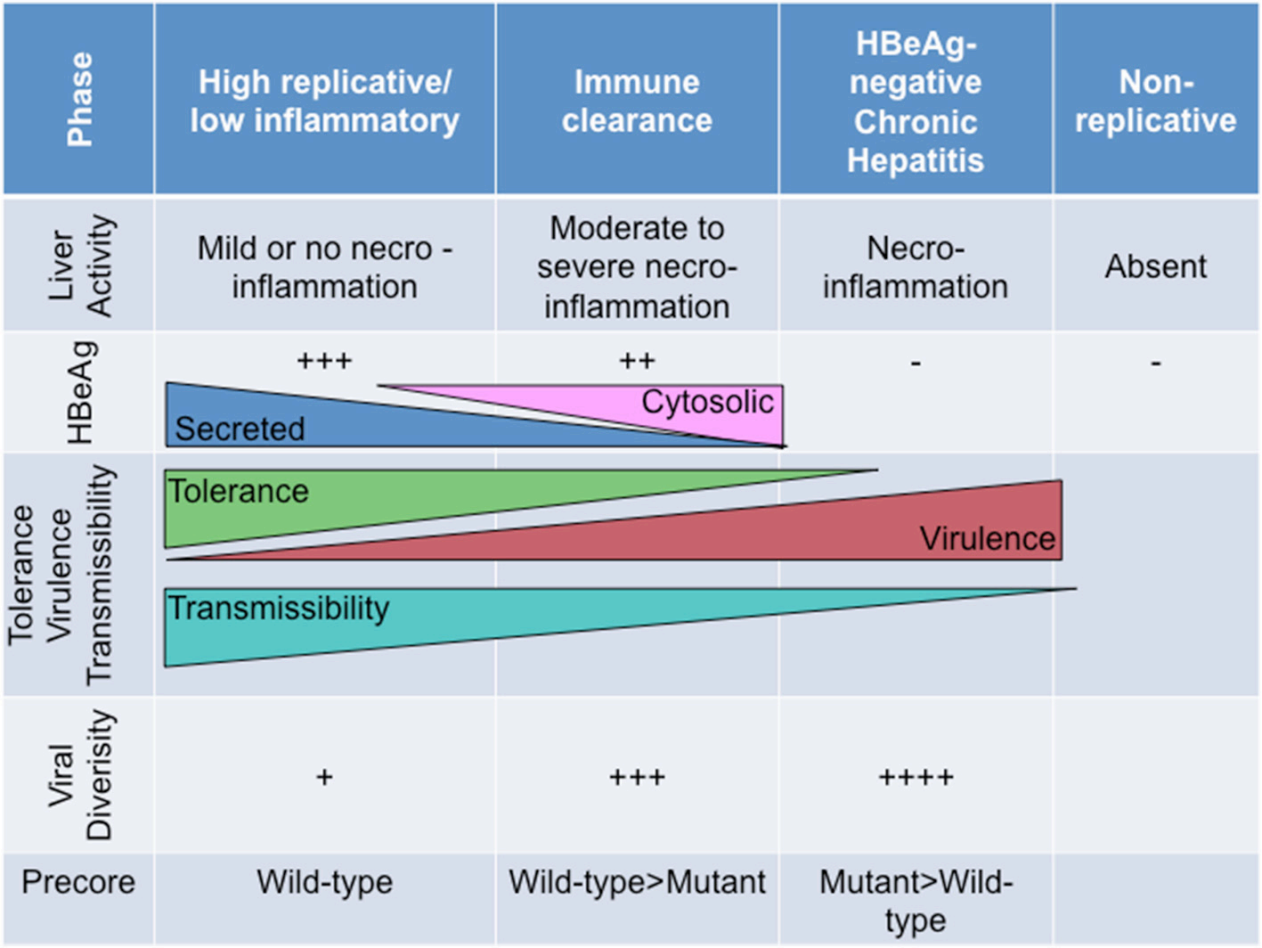
The relationship of HBeAg expression and precore region diversity to the natural history of HBV infection and its effect on tolerance, virulence and transmissibility of HBV.

Broadly, HBeAg-negativity is preferable at the individual host level whereas HBeAg-positivity is more favourable at the population level (Milich and Liang, 2003). As the natural course of HBV infection progresses from the HBeAg-positive (high replicative/low inflammatory) phase to the HBeAg-negative (chronic hepatitis) phase, T cell tolerance is ending as HBeAg expression changes from its secreted soluble to the cytosolic form. As a immunotolerogen and a decoy, secreted HBeAg is beneficial during the early phases of the infection, whereas the cytosolic form represents a liability considering that this form is processed and presented to MHC class I. Furthermore, HBeAg expressed *in vivo* is superior to HBcAg as a target, directly, or indirectly, of HBcAg/HBeAg-specific CTLs (Frelin et al., 2009). This differential CTL recognition of HBeAg, expressed by wild-type virus, may result in the preferential elimination of wild-type relative to HBeAg-negative HBV (Frelin et al., 2009). As a consequence, viral diversity increases during the course of infection, with the emergence of strains defective in HBeAg expression, which can escape the immune response and persist albeit at the cost, in some cases, of reduced replication, decreased transmissibility and increased virulence (Figure 5). Thus, this adaptive evolution of HBV during the course of HBeAg-seroconversion can be considered *short-sighted* when it limits the ability of the virus to transmit to new hosts, either by reducing per contact transmissibility or reducing contact rate of infected hosts because of increased virulence that can result in death (Lythgoe et al., 2017). The increased virulence can also be a result of the loss of immune tolerance. The ability of the different genotypes/subgenotypes to lead to HBeAg seroconversion can influence the degree of short-sighted evolution of HBV.

## Relationship of genotypes and subgenotypes to short-sighted evolution

As discussed above, mutations in the BCP/precore region, can affect HBeAg expression at the transcriptional, translational and post-translational level, and these mutations do not develop in all genotypes/subgenotypes at the same frequency (Table 1, Figures 3, 4). Subgenotype A2 and genotype H, develop only mutations 1762T/1764A, which affect transcription of precore mRNA and result in decreased levels of HBeAg (Buckwold et al., 1996; Baptista et al., 1999), without switching off HBeAg expression. Thus, these (sub)genotypes, which have been shown to have a relatively high frequency of HBeAg-positivity (Tanaka et al., 2004) are expected to have a long HBeAg high replicative, low inflammatory phase. This allows HBV to be transmitted sexually since it can remain asymptomatic allowing maturity for sexual activity and a healthy host (Araujo et al., 2011) before the development of cirrhosis and HCC. In fact, in a study carried out in the USA, genotype A (subgenotype A2 is predominates over A1 in the USA) was predominantly transmitted via sexual (82%) or parenteral (54%) routes compared to genotype B and C, which were transmitted vertically (Chu et al., 2003). On the other hand, the persistence of 1762T/1764A mutant expressing HBeAg, even at relatively low levels, means that the hepatocytes expressing HBeAg will be targeted, leading to liver injury and consequent development of cirrhosis and HCC much later in life. A high prevalence of 1762T/1764A has been detected in HBV strains from HCC patients compared to asymptomatic carriers of HBV (Baptista et al., 1999).

G1896A is the classical HBeAg-negative mutation that affects the translation of HBeAg by introducing a stop codon that truncates the HBeAg precursor and HBeAg is not expressed. Genotypes/subgenotypes with 1858T can develop the G1896A HBeAg-negative mutants (Table 1) and develop HBeAg-negative chronic hepatitis. Loss of HBeAg may be a sign of short-sighted evolution because there is loss of tolerogenic ability of HBeAg, HBeAg-negative virions are less transmissible and cannot establish chronic hepatitis. Establishment of chronic HBV infection following perinatal transmission requires wild-type HBV, whereas transmission of mixed wild-type/G1896A quasipecies leads to early immune elimination and resolution of the acute infection (Raimondo et al., 1993). G1896A occurs more frequently in genotype D than in genotype E strains (Figure 4), despite both having 1858T. This means that women of childbearing age infected with genotype E will remain HBeAg-positive for longer, allowing for mother-to-child transmission, which has resulted in high prevalence and geographical restriction of genotype E to Africa and among African emigrants to other regions (Mulders et al., 2004). Vertical transmission is advantageous in restricted human populations (Li et al., 2017). The higher HBeAg-positivity seen in individuals infected with genotype E compared to those infected with genotype D could confer tolerance and less serious clinical manifestations than genotype D (Yousif et al., 2013). Genotype E was found to prevail in Sudanese blood donors (Mahgoub et al., 2011), whereas the liver disease patients were infected with genotype D (Yousif et al., 2013). High ratios (>50%) of G1896A in the HBeAg-positive phase in individuals infected with genotype D are accompanied by persistently high viraemia and ALT elevation after anti-HBe seroconversion and a higher risk of cirrhosis (Chu et al., 2002).

Genotype G is a HBeAg-negative strain because of a premature stop codon at position 2 of the precore precursor protein and G1896A. Although genotype G is replication competent on its own (Li et al., 2007), it is always found as a co-infection with either subgenotype A2 or genotype H because the absence of the immunotolerogenic properties of HBeAg does not allow it to establish a persistent infection (Li et al., 2007). Like subgenotype A2 (Figure 3), genotype H develops 1896A infrequently and therefore can supply HBeAg in *trans* following co-infection. Genotype G can be transmitted and propagated without the helper virus and has been shown to replace genotype A in the HBeAg-negative phase of infection (Li et al., 2007) but requires the presence of helper virus expressing HBeAg in the early phases of infection in immunocompetent hosts (Kato et al., 2002). The absence of immunomodulatory HBeAg means that this genotype G alone can only infect immunocompromised individuals (Li et al., 2007). This is the reason why this genotype is relatively rare and is transmitted by high risk transmission chains including males-who-have-sex-with-men (MSMs), intravenous drug users (IVDUs) and blood transfusions.

Subgenotype A1 is the only strain that develops G1862T and HBV strains with G1862T have been isolated from tumorous but not from adjacent non-tumorous liver tissue (Kramvis et al., 1998). Thus, patients, infected with subgenotype A1 with the characteristic suboptimal Kozak sequence preceding the precore start codon, together with 1762T/1764A and 1862T mutant, would have severely decreased levels of HBeAg. The reduction or absence of HBeAg in the serum would result in the loss of immunotolerance and in the immune response being directed to the hepatocytes because of the reduction of soluble HBeAg, which acts as a decoy. This together with the increased ER stress, can result in liver damage, thus contributing to the higher hepatocarcinogenic potential of this subgenotype (Kramvis and Kew, 2007). In individuals infected with subgneotype A1, HCC develops 6.5 years earlier than in individuals infected with other (sub)genotypes (Kramvis and Kew, 2007). The early HBeAg seroconversion and the development of HCC before the age of 30, means that this subgenotype is characterized by the highest degree of short-sighted evolution as its mode of transmission is limited to early horizontal transmission and a short HBeAg-positive phase. This could be the reason why it is the only subgenotype, which can develop the unique mutations that affect HBeAg expression, Kozak 1809-1812 and G1862T at the translational and post-translational levels, respectively.

## Knowledge gaps and future studies

There is a paucity of studies on the natural history of subgenotype A1 of HBV, which prevails in Africa as opposed to subgenotype A2 found outside Africa. Moreover, there are no studies on the immune response to this particular strain of HBV. Further studies on the infection by this subgenotype in ethnic groups of non-African descent are important to determine whether the host genetic background influences its natural history. A relatively high prevalence of subgenotype A1 was found in HCC patients in southern India, and similarly to African studies, there was an association of subgenotype A1 with HCC and its development at a younger age (Gopalakrishnan et al., 2013). These studies are important and relevant considering the recent human migrations from Africa, which can lead to the dispersal of the strain globally.

## Conclusion

HBV is a very successful, highly evolved virus for a number of reasons. It has evolved in such a way that it can be transmitted by various modes, *in utero*, perinatally, horizontally early in life and sexually. Various (sub)genotypes have preferable mode/s of transmission. HBV can establish chronic infection and even though this may have serious consequences, including the development of cirrhosis, and HCC that may limit mobility and hence sexual transmission, these arise later in life long after the perinatal and neonatal transmission of the virus. Loss of HBeAg may be a sign of short-sighted evolution because there is loss of tolerogenic ability of HBeAg and HBeAg-negative virions are less transmissible. The different genotype/subgenotypes exhibit different degrees of short-sighted evolution.

## Author contributions

AK conceptualized and wrote the first draft of the paper, carried out mutation analysis. DP contributed to the writing and editing of the paper. E-GK carried out the phylogenetic analyses and prepared Figures 1, 2. AH contributed to the writing and editing of the paper. All authors listed have made a substantial, and intellectual contribution to the work, read and approved the final manuscript for submission.

### Conflict of interest statement

The authors declare that the research was conducted in the absence of any commercial or financial relationships that could be construed as a potential conflict of interest.
